# Combination of IL-33 with PD-1 blockade augment mILC2s-mediated anti-tumor immunity

**DOI:** 10.1007/s00262-023-03580-7

**Published:** 2024-03-02

**Authors:** Jiawei Yue, Hui Guo, Peng Xu, Jinhong Ma, Weifeng Shi, Yumin Wu

**Affiliations:** 1 Department of Orthopaedics, The Third Affiliated Hospital of Soochow University, Changzhou, 213003 Jiangsu China; 2 Department of Laboratory Medicine, The Third Affiliated Hospital of Soochow University, Changzhou, 213003 Jiangsu China; 3 Jiangsu Key Laboratory for Carbon-Based Functional Materials and Devices, Institute of Nano and Soft Materials (FUNSOM) College of Nano Science &Technology (CNST) Suzhou, Jiangsu, 215123 China

**Keywords:** Group 2 innate lymphoid cells, Interleukin-33, Cancer immunotherapy, Mature ILC2, Programmed cell death protein-1

## Abstract

**Background:**

Group 2 innate lymphoid cells (ILC2s) represent one of the main tissue-specific innate lymphoid cell populations, which are key drivers of cytokine secretion in their occupational niche. However, the precise involvement of ILC2s in cancer immunity and their potential impact on immunotherapeutic approaches remain poorly understood.

**Methods:**

The proportion of ILC2s originating from various tissue sources were quantified through flow cytometry, along with the determination of CD4^+^ T cell and CD8^+^ T cell percentages. Flow cytometry was also employed to assess IFN-γ production and programmed cell death protein-1 (PD-1) expression in T cells. Immunohistochemistry was utilized to detect IL-33 expression in tumor tissues, while immunofluorescence was employed to confirm the infiltration of ILC2s in both murine and human tumor tissues.

**Results:**

In this study, we provide evidence that intra-tumoral ILC2s in lung adenocarcinoma (LUAD) exist in a quiescent state. However, the activation of intra-tumoral ILC2s is induced by IL-33 specifically in a natural ILC2s (nILC2, ST2^+^KLRG1^−^) phenotype. Considering the pivotal role of PD-1 in cancer immunotherapy and its immunoregulatory functions, we investigated the synergistic effects of IL-33 and anti-PD-1 and found that their combination enhances anti-tumor immunity and improves the efficacy of immunotherapy. Moreover, this combination leads to the upregulation of activated mature ILC2s (mILC2, ST2^+^KLRG1^+^) phenotype, thereby highlighting the activated ILC2s as a novel enhancer of the immunoregulatory properties of anti-PD-1.

**Conclusions:**

Collectively, these findings underscore the significance of ILC2s and their contribution to the anti-tumor response in the context of cancer immunotherapy. Consequently, the simultaneous targeting of ILC2s and T cells represents a potentially promising and widely applicable strategy for immunotherapeutic interventions.

**Supplementary Information:**

The online version contains supplementary material available at 10.1007/s00262-023-03580-7.

## Introduction

Lung cancer is a highly aggressive form of cancer with high rates of morbidity and mortality [1]. If diagnosed at an early stage, effective treatment can be obtained, but 20% of patients with late-stage lung disease do not respond to treatment [1]. Tumor invasion and metastasis are not solely dependent on cancer cells, but are closely associated with the composition of immune cells in the tumor microenvironment (TME) [2]. Cancer cells and their secreted cytokines orchestrate the establishment of a pro-tumoral microenvironment, characterized by immune evasion mechanisms that impede effective immune surveillance [3]. Notably, several immune cells also participate in the formation of the pro-tumoral immune microenvironment, further accentuating the suppressive of TME, thereby posing significant challenges in the realm of tumor prevention and treatment. Therefore, it is necessary to explore the mechanism of this phenomenon.

Increasing evidence suggests that innate lymphoid cells (ILCs) are one of the major immune cell types in the TME that need to be given more attention [4, 5]. Unlike dendritic cells (DCs), macrophages, and T lymphocytes, innate lymphoid cells (ILCs) do not express specific lineage markers, and are therefore often defined as lineage-negative [6]. They often reside in mucosal tissues, including the lung, gut, and skin, however, can migrate [7]. They were divided into four groups, and their nomenclature was based on the nomenclature of helper T cells. (1) group 1 ILCs (ILC1s), producing interferon g (IFN-γ) [8]; (2) group 2 ILCs (ILC2s), producing type 2 cytokines, in particular, IL-5 and IL-13 [9]; (3) group 3 ILCs (ILC3s), producing IL-17 and/or IL-22 [10]; and regulatory ILCs (ILCreg), producing IL-10 and TGFβ [11]. Among all these groups, group 2 Innated Lymphoid cells (ILC2s) have caught the eyes of more and more researchers and studied their potential role in various diseases for their large number and wide distribution. ILC2s exert their function through their effector function cytokines when activated by epithelial-derived alarmin, including interleukin (IL-) 25, IL-33, and Thymic Stromal Lymphopoietin (TSLP) [12]. ILC2s are also poised to play a significant role in tumorigenesis due to their unique occupational niche in mucous membrane tissue [13] and more than 80% of all tumors originate from epithelial tissues [14]. Recently, several research groups have reported the infiltration of innate lymphoid cells (ILCs) within melanoma tumors. Specifically, ILC2s have been shown to co-recruit eosinophils through the production of cytokines such as IL-5 and granulocyte–macrophage colony stimulating factor (GM-CSF), thereby providing protection against primary tumor development [15]. Although the involvement of ILC2s in Th-2 inflammatory diseases has been extensively studied and established, their role in cancer has remained a subject of controversy, necessitating further investigation.

Currently, ILC2s can be classified into three subgroups: (1) natural ILC2s (nILC2s), which are constitutively present in tissues under steady-state conditions, (2) inflammatory ILC2s (iILC2s), which can be recruited from the circulation and lymphoid organs in response to inflammatory stimuli [16], (3) mature ILC2s (mILC2s), among which nILC2s are defined as ST2^+^KLRG1^–^ cells, while iILC2s are characterized by the absence of ST2 and high expression of KLRG1, mILC2s were defined as KLRG1^+^ST2^+^ [17]. Given the heterogeneity and contentious nature of ILC2s within the TME, it is imperative to discern and validate the specific subsets that infiltrate the TME. Emerging evidence suggests that ILC2s can express both PD-1 and PD-L1 upon activation by the cytokine IL-33 [18]. Consistent with T, NK, and B cells, the expression of PD-1 on ILC2s has been shown to substantially diminish cell proliferation, viability, and effector capacity. Blocking PD-1 signaling has been found to enhance the overall function and abundance of ILC2s, leading to heightened anti-tumor effects within the TME [19], thus, it is crucial to investigate the specific subset of ILC2s that contributes to this anti-tumor functionality within the TME.

In this report, we demonstrate a notable increase in the proportion of ILC2s within tumor tissue when compared to spleen and lung tissue. This increase in tumor-infiltrating ILC2s is accompanied by a dampened immune response mediated by CD4+ T/CD8+ T cells. However, co-administration of IL-33 and PD-1 blockade leads to a significant augmentation in the percentage of ILC2s, particularly iILC2s, and enhances the anti-tumor response. These findings shed further light on the immunobiology of ILC2s in the context of anti-tumor activity and its potential role in traditional immune checkpoint therapy. Importantly, our research holds promise for advancing our understanding of the therapeutic value of ILC2s in clinical cancer treatment.

## Materials and methods

### Mice

C57BL/6J mice were purchased from the Cavens Biogle Model Animal Research Co. Ltd. IL-33^−/−^ (C57BL/6-*il33*^*em1Smoc*^) mice were generated in the Shanghai Model Organisms Center, Inc. All the animals were bred in our animal facility at the Soochow University. Experimental protocols were approved by the ethical committee of Soochow University (Suzhou, China) and all conform to the provisions of the Declaration of Helsinki.

### Cancer cell culture

The murine lung adenocarcinoma LLC (CRL-1642, ATCC) was obtained from the ATCC. The cell line was cultured in DMEM/F12 medium (Life Technologies) with 10% heat-inactivated FBS and 1% penicillin and streptomycin in 5% CO_2_ at 37 °C.

### In vivo experiments and tissue preparation

For subcutaneous tumors, tumor cells (LLC cells, 1 × 10^6^ cells) were resuspended in sterile PBS (Fisher Scientific) and implanted subcutaneously. Tumor dimensions were measured using calipers every 2–3 days, and tumor volume was calculated using the formula: volume = 1/2 length × width^2^. Mice were euthanized if the tumor size reached 1500 mm^3^ or the tumor became ulcerated. For the orthotopic lung cancer model, the first 4-mm incision was made on the left side of the chest. The soft adipose tissue was then carefully removed to expose the sternum and intercostal space, making the left lobe more visible. During surgery, the cell mix (LLC-LUC, 5 × 10^5^ cells) was injected directly into the lungs. Surgical screws were removed 1 week after injection. Bioluminescence imaging (BLI) was performed 7 days after injection using the IVIS imaging system (Xenogen). Briefly, after anesthesia, the mice were intraperitoneally injected with D-luciferin (150 mg/kg, intraperitoneally). Imaging was performed 2 min after 10 min of injection. The mice were sacrificed at the specified time point and examined by histology or flow cytometry. Briefly, the lungs and tumors were perfused with PBS, mechanically dissociated and incubated in 0.5 mg/ml Liberase TM (Roche Diagnostics Corporation, Indianapolis, IN) and DNAse I (Roche Diagnostics Corporation, Indianapolis, IN) at 37 °C for 1 h. Digestion was then quenched with fetal bovine serum (FBS, Life Technologies), and cells were filtered through a 70 μm nylon cell filter (Falcon, Fisher Scientific). Spleens were mechanically isolated and filtered through 70 μm nylon cell strainers (Falcon, Fisher Scientific) using PBS with 1% FBS, followed by RBC lysis (RBC lysis buffer, Thermo Fisher Scientific). Mouse Fc receptors were blocked with anti-16/32 antibody (1 μg per 1 × 10^6^ cells; Biolegend, CA, USA).

### IHC

After deparaffinization, to inhibit endogenous peroxidase, 3% hydrogen peroxide was used to incubate tumor sections, to heat-induce epitope retrieval, microwave treated them in 10 mmol/L citrate buffer (pH 6.0). Then the sections were incubated with rabbit anti-mouse IL-33 for 60 min at room temperature (1:200, Abcam). Incubation was done in a broad antibody enhancer and polymer–horseradish peroxidase, and then the sections were stained with the DAB Detection System. Finally, slides were counterstained with hematoxylin.

### Recombinant IL33 administration and PD-1 blockade

For rmIL33, mice were then intravenously (*i.v.*) injected with 1 μg recombinant mouse IL-33 (Sigma) in sterile PBS every 2 days. For PD-1 blockade, mice were intraperitoneally (*i.p.*) injected with 200 μg anti-PD-1 (Bio X cell, CA, USA) every 2 days. A partial response was defined as a temporary reduction in tumor volume but subsequent regeneration following continued anti-PD-1 therapy. When the tumor size did not decrease, and continuous resistance to PD-1 was defined as drug resistance.

### Flow cytometry

The single-cell suspensions were stained in the darkness at 4 °C using antibody cocktails, washed, and analyzed on a FACS Canto II (BD Biosciences). Mouse ILCs were defined as live, CD45+, lineage- (CD3, CD45R, CD11b, CD19, Ly-G6), CD127^+^, CD25 cells as previously described [20]. For ILC2 subsets, ILC2s were further stained with ST2 and KLRG1. Mouse immune cells were defined as follows: ILC2s = live, CD45+, lineage-CD25+, CD127+ cells; nILC2s = ST2+KLRG1-ILC2s; iILC2s = ST2+KLRG1- ILC2s; mILC2s = ST2+KLRG1 + ILC2s; T cells = live, CD45+CD3+; CD8^+^ T cells = live, CD45+CD3+CD8+; CD4^+^ T cells = live, CD45+CD3+CD4+; regulatory T cells = live, CD45+CD3+CD4+FoxP3+; Murine cells were stained with the following antibodies: from Thermo Fisher Scientific, CA, USA, FITC-lineage (clone, 17A2, RA3-6B2, M1/70, TER-119, RB6-8C5), Alexa Fluor 700-IFN-γ (clone, XMG1.2); from Biolegend, CA, USA, APC-Cy7-CD45 (clone, S18009F), PE-Cy7-CD25 (clone, 3C7), PE-CD127 (clone, A7R34), Percp-cy5.5-KLRG1 (clone, 2F1/KLRG1), FITC-CD3 (clone, 145-2C11), PE-Cy7-CD4 (clone, GK1.5), Percp-cy5.5-CD8 (clone, 53–6.7), PE-Foxp3 (clone, MF-14), APC-PD-1 (clone, 29F.1A12).

To detect intracellular cytokine production, tumor single cell suspensions were stimulated in vitro with a cell-stimulating cocktail for 6 h (Thermo Fisher Scientific, CA, USA) at 37 °C. Cells were then surface-stained, fixed, permeabilized, and stained for cytokine production using the Fixation and Permeabilization Buffer Kit per the manufacturer’s recommendations (Thermo Fisher Scientific, CA, USA). To stain Foxp3, the samples were prepared using eBioscience™ Foxp3/Transcription Factor Staining Buffer Set (Thermo Fisher Scientific, CA, USA) after surfaced markers staining. The FACS data were analyzed with Flowjo10 software (version 7.6.5; Tree Star, Ashland, OR, USA), Gating strategies were shown in Supplementary Fig. 1A, B.

### Murine ILC2s isolation and in vitro culture

Murine ILC2s were isolated from total splenocytes on a FACS Aria II system. Briefly, the murine splenic cell suspension was incubated with FITC-lineage (CD3, CD45R, CD11b, CD19, Ly-G6) (Thermo Fisher Scientific, CA, USA, clone, 17A2, RA3-6B2, M1/70, TER-119, RB6-8C5), PE-CD127 (Biolegend CA, USA, clone, A7R34), and APC-Cy7-CD25 (Biolegend CA, USA, clone, 3C7), and then ILC2s were sorted by flow cytometry. Cells were stimulated with IL-2 (20 ng/ml) (R&D Systems, MN, USA) and IL-33 (10 ng/ml) (R&D Systems, MN, USA) for 72 h before use.

### Sample tissue microarrays

All tissues were collected at the Third Affiliated Hospital of Soochow University (Changzhou, China) following the study protocol. Informed consent was obtained for all patients. The study was performed in strict compliance and approval by the ethical committee of the Third Affiliated Hospital of Soochow University. All tumor samples were surgically resected for primary lung adenocarcinoma. Tumor and adjacent non-tumor tissues were fixed in formalin, and then constructed tissue microarrays (TMAs) (*n* = 30 tumors, 30 normal tissues). Those patients who were treated with neoadjuvant therapy were excluded.

### Immunofluorescence

TMAs were blocked with background Buster solution (Innovex) for 30 min, followed by avidin–biotin for 8 min. Human FITC-Lineage (CD2, CD3, CD14, CD16, CD19, CD56, CD235a), KLRG1 antibodies were applied at 4 °C overnight and followed by a Cy5-conjugated anti-mouse secondary antibody (Thermo Fisher Scientific, CA, USA). For mice, mouse FITC-Lineage (CD3, CD45R, CD11b, CD19, Ly-G6), GATA3 (Thermo Fisher Scientific, CA, USA), CD127 (Thermo Fisher Scientific, CA, USA) antibodies were applied at 4 °C overnight and followed by a PE-conjugated anti-rat (Thermo Fisher Scientific, CA, USA) and a Cy5-conjugated anti-rabbit secondary antibody (Thermo Fisher Scientific, CA, USA) according to the manufacturer's instructions. After staining, counterstained the TMAs with DAPI (Sigma Aldrich) for 10 min. For quantification, each nucleus was segmented using the DAPI channel after proper processing and background subtraction. Counter the number of cells with a particular combination of markers. KLRG1 expressing Lineage^−^ cells were defined as Lineage^−^ KLRG1^+^ nucleated cells. For each patient, the frequency of Lineage^−^ KLRG1^+^ was calculated in triplicate cores, and then the average frequency of triplicate cores was measured to calculate the final cells.

### Statistics

Data are expressed as median. Comparisons between the two groups were performed using an unpaired t-test. Multi-group comparisons across multiple time points were performed using two-way ANOVA tests. The survival curves were compared by bilateral log-rank test. When *p* < 0.05 was considered a significant difference. Statistical analyses were performed using Prism 7.0 (GraphPad Software).

## Results

### ILC2s infiltrating in tumor tissues compared with normal tissues

**Fig. 1:**
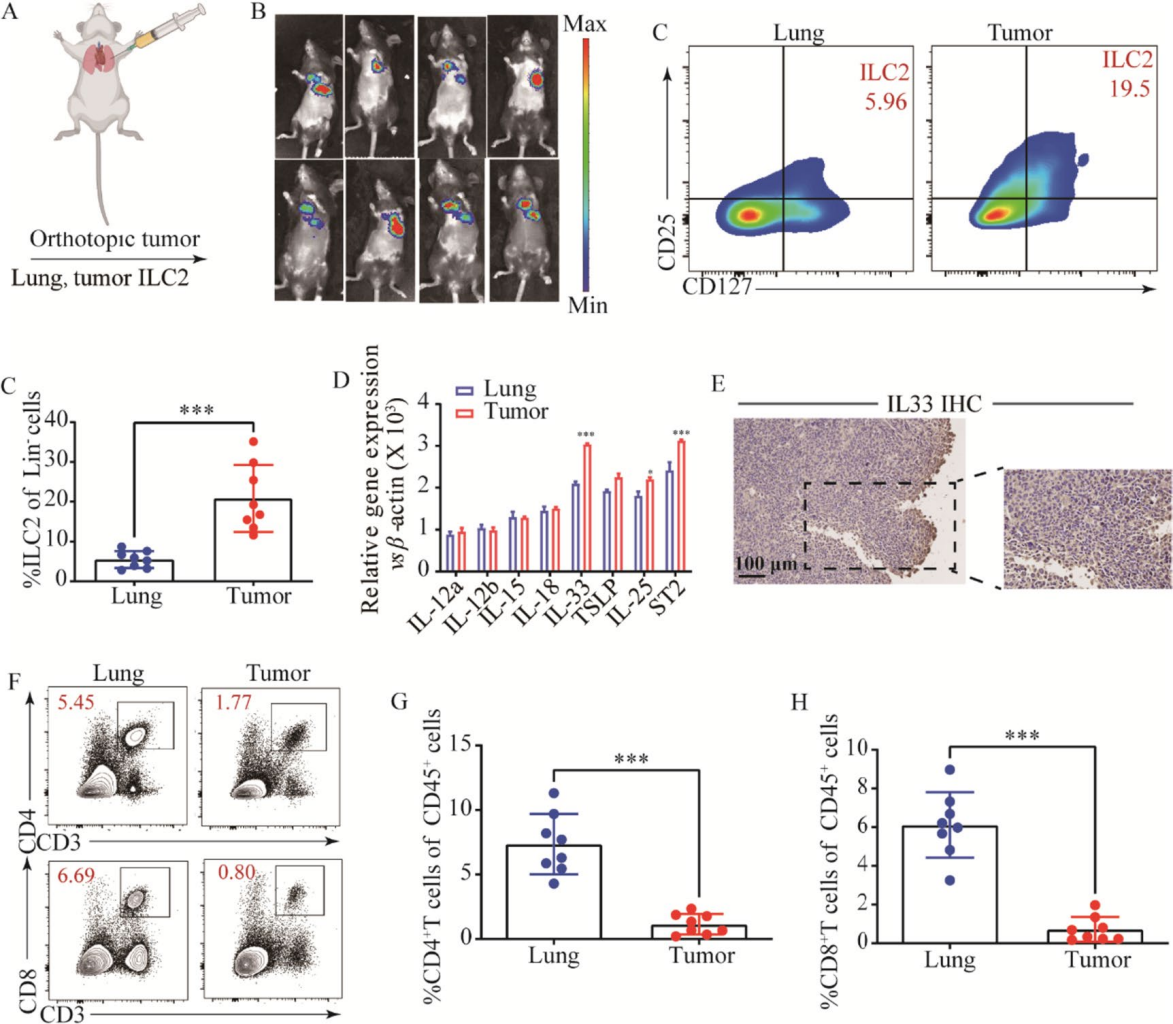
ILC2s infiltrating in murine lung adenocarcinoma tumor tissue. **A** Orthotopic LUAD tumor model. **B** In vivo imaging images of orthotopic lung cancer. **C** and **D** Gating (**C**) and frequency (**D**) of ILC2s in orthotopic LUAD tumor-bearing mouse.** E** Relative mRNA expression of ILC1- (IL-12a, IL-12b, IL15, IL18), ILC2s- (IL25, IL33, TSLP), and the ST2 in lung tissues, and tumor tissues of orthotopic LUAD tumor-bearing mice. **F** IL-33 protein expression of IL-33 in tumor tissues of orthotopic LUAD tumor-bearing mice. Gating and phenotype of CD4^+^ T cell and CD8^+^ T cell (**G**) in lung tissues and tumor tissues. Frequency of CD4^+^ T cell (**H**), and CD8^+^ T cell (**I**) in lung tissues and tumor tissues of orthotopic LUAD tumor-bearing mice. Data were pooled from ≥ 2 independent experiments with *n* = 8/group; *n* and data points denote individual mice analyzed separately. *P* values were determined by unpaired t test. *P < 0.05, **P < 0.01, ***P < 0.001

Considering the significant differences in TME among different cancer types, as well as the presence of distinct immune cell populations in certain organs [21], we initially established an orthotopic cancer mode to investigate whether ILC2 participates in the progression of lung adenocarcinoma (Fig. 1A, B). There was an elevated infiltration of ILC2s in the tumor tissues compared with para-carcinoma lung tissues (Fig. 1C, D). In pancreatic cancer, IL-33 has been further elucidated as an effector molecule that induces early tumor initiation and tumor transformation [22. This suggests that the enrichment of IL-33 in the TME provides a favorable milieu for the survival and expansion of ILC2. However, considering the distinct subtypes of ILCs, further investigations were conducted to ascertain whether factors conducive to the survival and expansion of ILC2s exist within the TME of LUAD. Therefore, we next investigated the expression levels of key factors involved in ILCs regulation, both in tumor tissues and adjacent non-cancerous tissues. We found that, in comparison to other inducers of ILCs such as ILC1s or ILC3s, ILC2s-inducer cytokines including IL-33, IL-25, and TSLP, along with their characteristic markers, are highly abundant in tumors (Fig. 1D). Next, we analyzed the most potent inducer IL-33 protein in tumor tissues, we found that IL-33 is widely expressed in tumor tissues (Fig. 1E). Worth noting is that through further correlation analysis, we found a positive correlation between the mRNA and protein expression levels of IL-33 and the infiltration of ILC2s (Supplementary Fig. 2B, C). Given the suppression of TME, we next analyzed the infiltration of CD4^+^ T cells and CD8^+^ T cells. Decreased proportion of CD4^+^ T cells and CD8^+^ T cells were found compared with para-carcinoma lung tissue (Fig. 1F–H). Collectively, these results demonstrate that the abundance of ILC2s in tumor tissues is higher relative to other tissues, and there are factors inducing ILC2s.

### Tumor-derived ILC2s were in the rest state

**Fig. 2:**
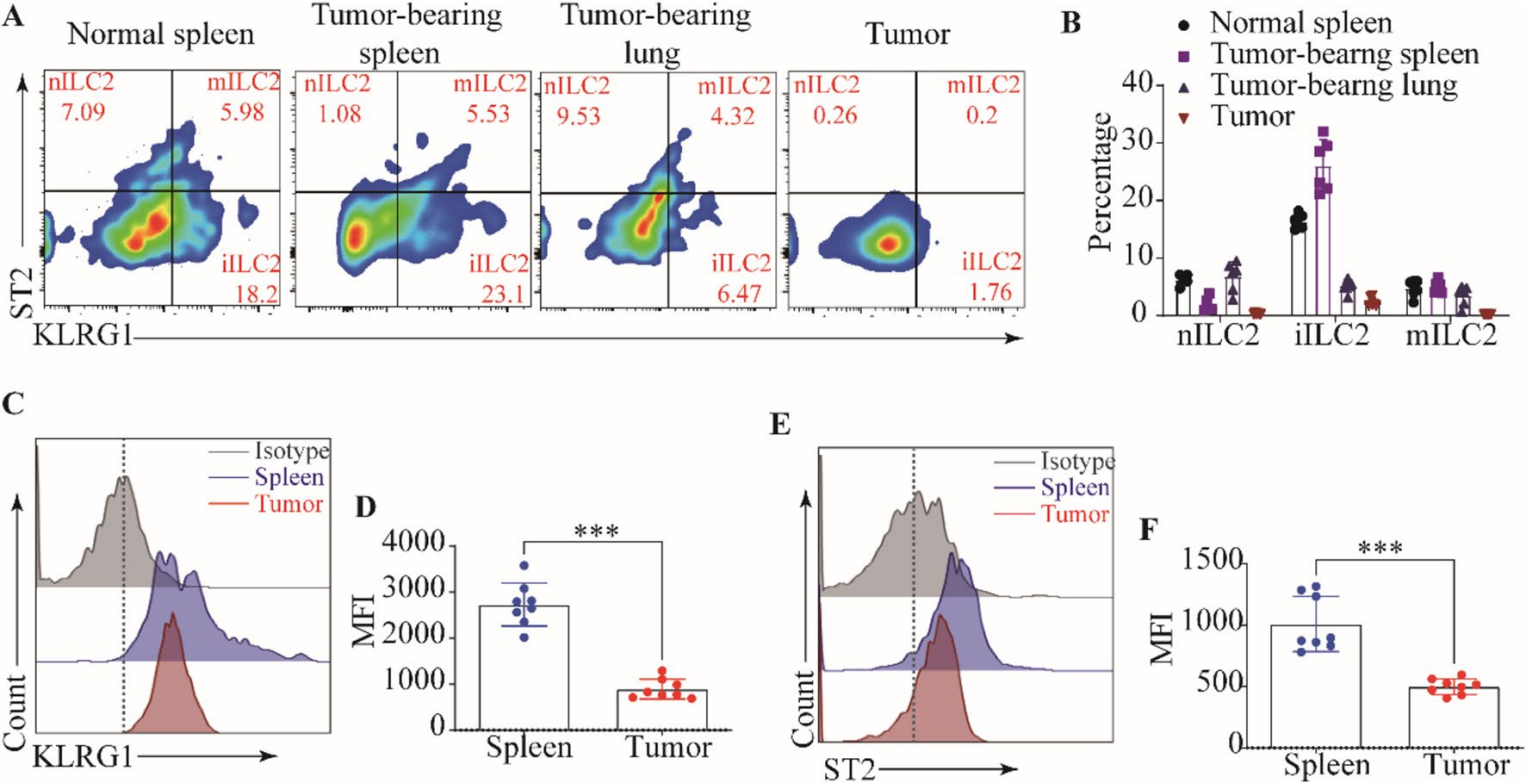
Tumor-derived ILC2s were in a rest state. **A** Gating and phenotypes of nILC2s, iILC2s, and mILC2s in the normal spleens, tumor-bearing spleens, tumor-bearing lungs, and tumor tissues of tumor-bearing mice. **B** Frequencies of nILC2s, iILC2s, and mILC2s in the normal spleens, tumor-bearing spleens, tumor-bearing lungs, and tumor tissues of tumor-bearing mice. The staining and MFI of KLRG1 (**C**) and ST2 (**D**) in ILC2s from tumor-bearing lungs, and tumor tissues of tumor-bearing mice were analyzed by flow cytometry. Data were pooled from ≥ 2 independent experiments with n ≥ 5/group; n and data points denote individual mice analyzed separately. *P* values were determined by unpaired t test. ***P < 0.001

Encouraged by the aforementioned results, we intend to further investigate the role of ILC2s in subcutaneous tumors (Supplementary Fig. 3A). As widely recognized, despite the abundance of immune cells in both the spleen and TME, it is noteworthy that immune cell infiltration and function within the TME are often suppressed [23, 24]. This inhibitory milieu affects even the same cell types, facilitating the evasion of tumor cells from immune surveillance mechanisms, thereby enabling their escape from immune-mediated eradication. Consequently, we proceeded to analyze the disparity in the population of ILC2s between the spleen and tumor tissue. The results revealed a significant upregulation in the proportion of ILC2s within tumor tissue compared to splenic tissue (Supplementary Fig. 3B, C). Furthermore, this upregulation of ILC2s was accompanied by a downregulation of CD4^+^ T cells and CD8^+^ T cells (Supplementary Fig. 3D–G). Due to the exhaustion state frequently observed in infiltrating T cells within the TME, we then assessed the expression of PD-1 on CD4^+^ T cells and CD8^+^ T cells. elevated PD-1 was found in CD4^+^T cells and CD8^+^T cells in the tumor tissues compared with in the spleens (Supplementary Fig. 3H–K). As ILC2s have three subsets, we hypothesized that ILC2s in TME would be tissue-specific. To test this, we further detected KLRG1 and ST2 expression in ILC2s residents in the normal spleen, tumor-bearing spleen, tumor-bearing lung tissue, and tumor tissue. Our results showed that tumor-derived ILC2s were in the rest state with nearly undetectable KLRG1 and ST2 compared with spleen-, and lung-derived ILC2s (Fig. 2). We found that in the tumor tissues of tumor-bearing mice, nILC2s (ST^+^KLRG1^−^ ILC2s), iILC2s (ST2^−^KLRG1^+^ ILC2s), and mILC2s (ST2^+^KLRG1^+^ ILC2s) were almost undetectable and most of the ILC2s were in a quiescent state (Fig. 2A, B). Subsequently, the comparison of the mean fluorescence intensity (MFI) values of ST2 and KLRG1 expressed by ILC2s from the spleens and tumor tissues of the tumor-bearing mice also confirmed this. The results demonstrated that the expression levels of KLRG1 and ST2 in ILC2s derived from tumor tissue were comparatively lower when compared to ILC2s derived from splenic tissue **(**Fig. 2C–F). These findings suggest a potential correlation between the expression of KLRG1 and ST2 and the functional properties of ILC2s. Consistent findings were observed in human samples, where a reduced infiltration of Lin^−^ KLRG1^+^ cells was detected in tumor tissues compared to para-carcinoma tissues (Supplementary Fig. 4A–D). These observations suggest a diminished presence of KLRG1^+^ Lin^−^ lymphocytes, including ILC2s, within human tumor tissues. Collectively, these findings suggest that while the abundance of ILC2s in tumor tissues is higher, a majority of these cells exist in a quiescent state characterized by diminished expression levels of ST2 and KLRG1.

### Lost host-derived IL-33 promotes tumor growth

**Fig. 3:**
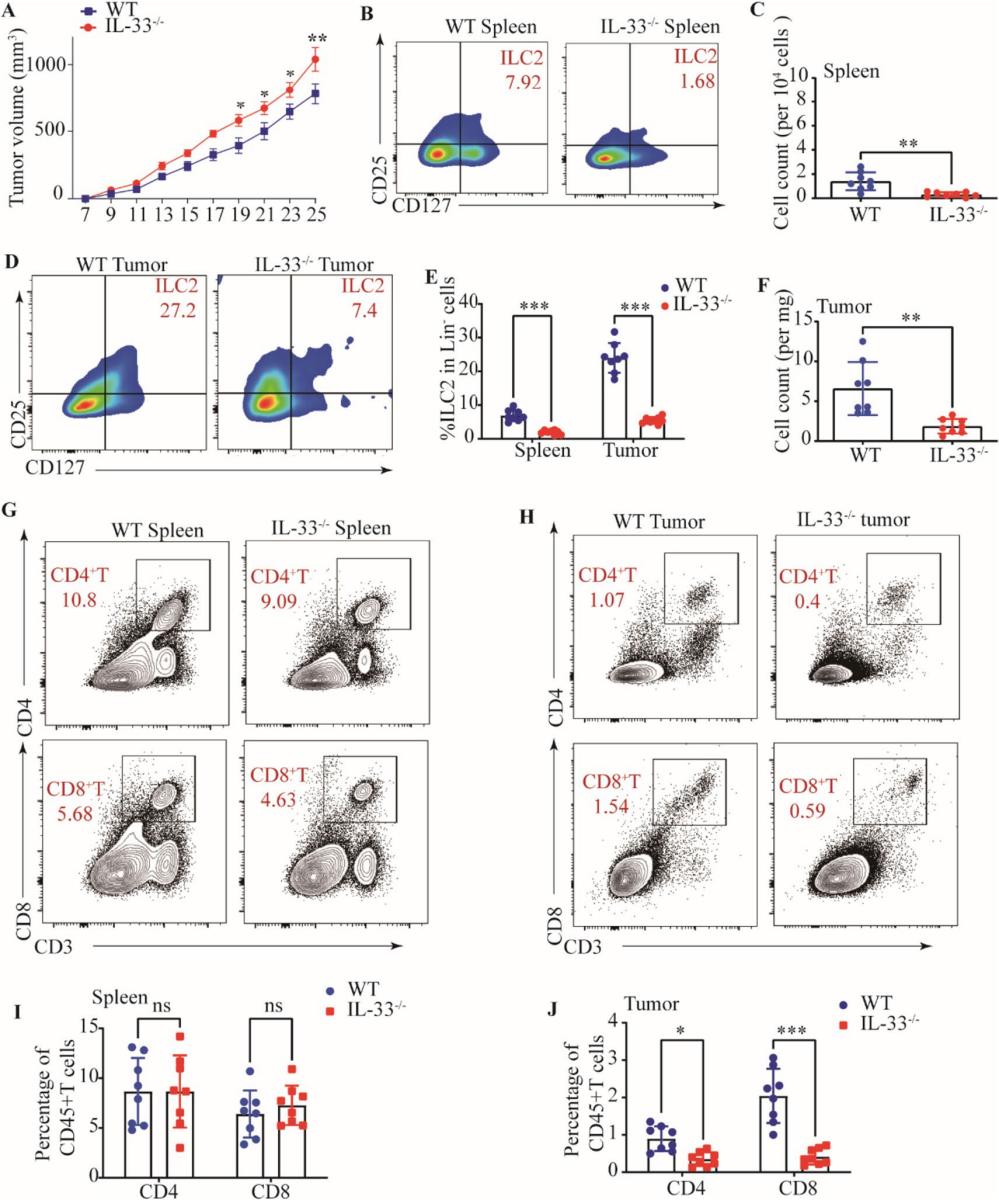
Lost host-derived IL-33 promotes tumor growth. **A** Tumor volumes of tumors in WT and IL-33^−/−^ mice. Gating (**B**) and number (**C**) of ILC2s in the spleens of WT and IL-33^−/−^ tumor-bearing mice. **D** Gating of ILC2s in the tumor tissues of WT and IL-33^−/−^ tumor-bearing mice. **E** Frequencies of ILC2s in spleens and tumor tissues of WT and IL-33^−/−^ tumor-bearing mice. **F** Numbers of ILC2s in tumor tissues of WT and IL-33^−/−^ tumor-bearing mice. Gating and phenotypes of CD4+ T cell and CD8+ T cell in the spleens (**G**) and tumor tissues (**H**) of WT and IL-33^−/−^ tumor-bearing mice. Frequencies of CD4+ T cell and CD8+ T cell in the spleens (**I**) and tumor tissues (**J**) of WT and IL-33^−/−^ tumor-bearing mice. Data were pooled from ≥ 2 independent experiments with *n* = 8/group; n and data points denote individual mice analyzed separately. *P* values were determined by two-way analysis with multiple comparisons (**E**, **I**, **J**), unpaired t test (**C**, **F**). ***P < 0.001, **P < 0.01, *P < 0.05, ns, no significant

Motivated by the aforementioned findings and recognizing the potent capacity of IL-33 to induce activation of ILC2s, we subsequently employed IL-33 to delineate and compare the impacts of IL-33 deficiency on the dynamic patterns of tumor growth. In contrast to their WT counterparts, mice lacking IL-33 (IL-33^−/−^) demonstrated significantly increased tumor size and accelerated tumor growth (Fig. 3A). We then proceeded to verify whether these differences were due to altered ILC2 development. We initially corroborated a substantial reduction in both the proportion and absolute count of ILC2s within the spleens of IL-33^−/−^ tumor-bearing mice, in stark contrast to their wild-type tumor-bearing counterparts (Fig. 3B, C, E). Meanwhile, we observed a significant decline in the frequency and quantity of ILC2s within the tumor tissues of IL-33^−/−^ tumor-bearing mice, in comparison to WT tumor-bearing mice (Fig. 3D–F and Supplementary Fig. 5A–D). Consistently, IL-33^−/−^ tumor-bearing mice had lower frequencies of tumor-infiltrating CD4^+^ T cells and CD8^+^ T cells (Fig. 3H, J), while no consistent alterations were observed in the overall populations of CD4^+^ T cells and CD8^+^ T cells (Fig. 3G, I). These observations confirm that the absence of IL-33 does not impact T cell development, however, it highlights the crucial role of IL-33 in mediating anti-tumor effects through its close association with T cells. Collectively, these findings suggest that the absence of IL-33 within tumor tissues promotes an accelerated rate of tumor growth and is accompanied by a downregulation of adaptive immune responses, which may potentially be associated with the depletion of ILC2s.

### Exogenous rmIL-33 boosts nILC2s-mediated immune response

**Fig. 4:**
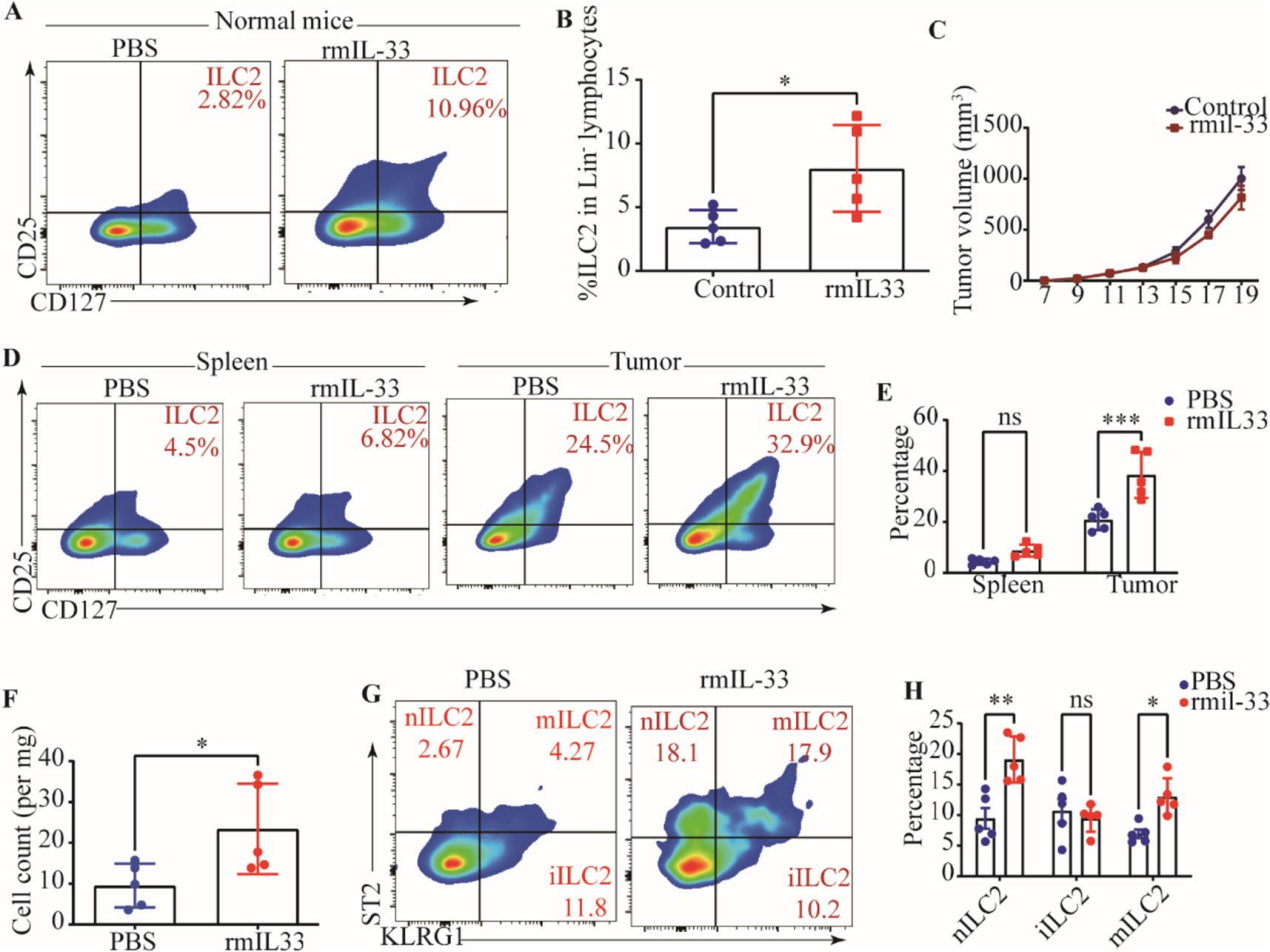
Exogenous rmIL-33 promotes enhanced nILC2s percentage.** A** Gating and phenotypes of ILC2s in the spleens of PBS and rmIL-33 treated normal mice. **B** Frequency of ILC2s in spleens of PBS and rmIL-33 treated normal mice. **C** Tumor volumes of PBS and rmIL-33 treated tumor-bearing mice. **D** Gating and phenotypes of ILC2s in the spleens and tumor tissues of PBS and rmIL-33 treated tumor-bearing mice. **E** Frequencies of ILC2s in the spleens and tumors of PBS rmIL-33 treated tumor-bearing mice. **F** Numbers of ILC2s in the tumors of PBS rmIL-33 treated tumor-bearing mice. **G** Gating and phenotypes of nILC2s, iILC2s, and mILC2s in the tumor tissue. **H** Frequencies of nILC2s, iILC2s, and mILC2s in tumors of PBS and rmIL-33 treated tumor-bearing mice. Data were pooled from ≥ 2 independent experiments with *n* = 5/group; n and data points denote individual mice analyzed separately. *P* values were determined by unpaired t test (**B**, **F**), two-way analysis with multiple comparisons (**E**, **H**). ***P < 0.001, **P < 0.01, *P < 0.05, ns, no significant

**Fig. 5:**
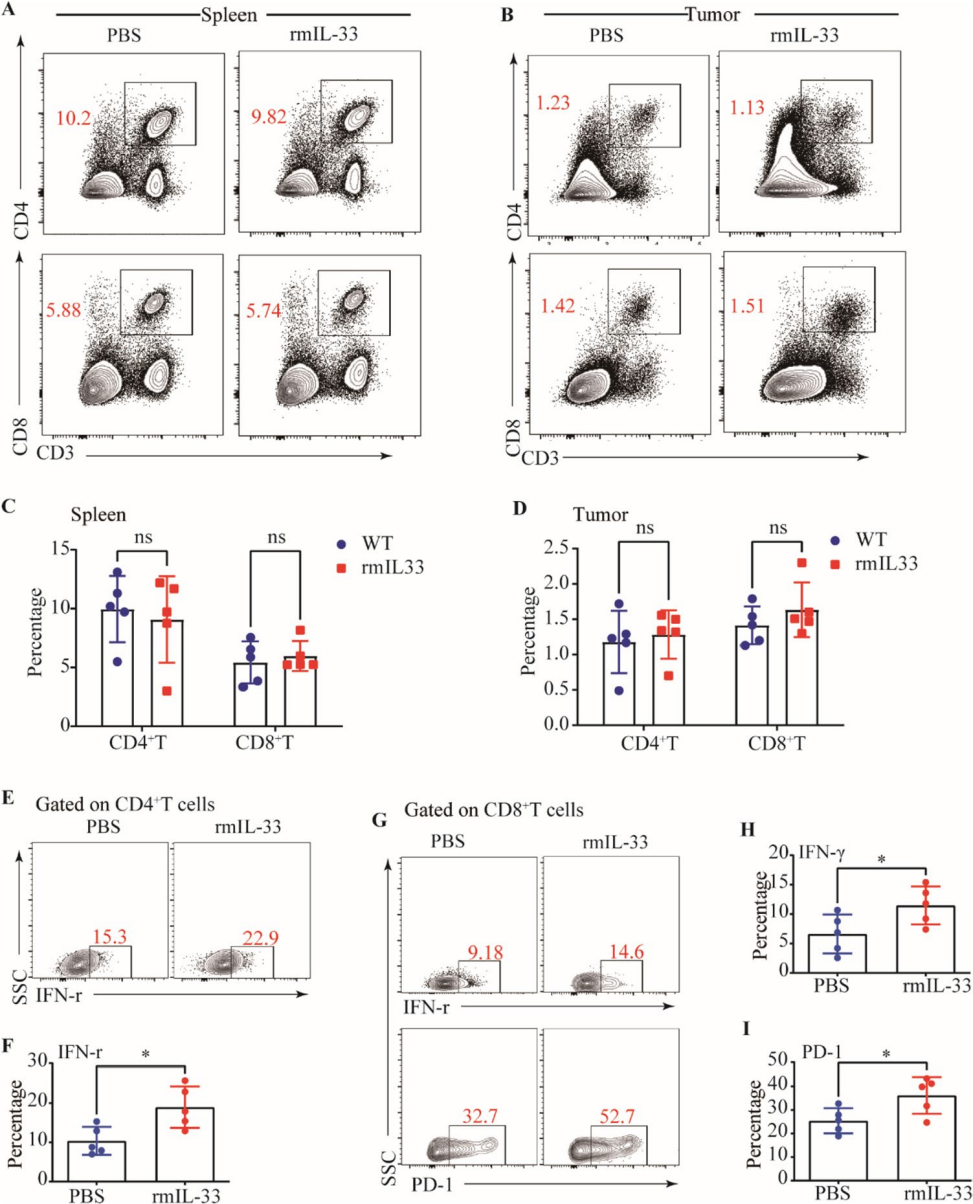
Exogenous rmIL-33 boosts intra-tumoral CD4^+^T cell and CD8^+^T cell cytokine capacity and PD-1 expression. Gating and phenotypes of CD4^+^T cell and CD8^+^T cell in the spleens (**A**) and tumor tissues (**B**) of PBS and rmIL-33 treated tumor-bearing mice. Frequencies of CD4^+^T cell and CD8^+^T cell in the spleens (**C**) and tumor tissues (**D**) of PBS and rmIL-33 treated tumor-bearing mice. Gating (**E**) and frequency (**F**) of IFN-γ of CD4^+^T cell in the tumor tissues of PBS and rmIL-33 treated tumor-bearing mice. **G** Gating and phenotypes of IFN-γ and PD-1 of CD8^+^T cell in the tumor tissues of PBS and rmIL-33 treated tumor-bearing mice. Frequencies of IFN-γ (**H**) and PD-1 (**I**) of CD8^+^T cell in the tumor tissues of PBS and rmIL-33 treated tumor-bearing mice. Data were pooled from ≥ 2 independent experiments with *n* = 5/group; n and data points denote individual mice analyzed separately. *P* values were determined by two-way analysis with multiple comparisons (**C**, **D**), unpaired t test (**F**, **H, I**). *P < 0.05, ns, no significant

Our previous findings have provided evidence that the deficiency of IL-33 leads to a depletion of ILC2s, which is accompanied by accelerated growth of tumors and impaired adaptive immune responses. Considering the impact of IL-33 on ILC2s, our subsequent investigation aimed to confirm the impact of exogenous rmIL-33 on ILC2s. We observed a significant enhancement in ILC2s-mediated immune response following rmIL-33 treatment, as evidenced by an increased proportion of global ILC2s in the spleens of normal mice (Fig. 4A, B). However, our findings indicated that the administration of rmIL-33 did not exhibit any preventive effects on tumor establishment and did not elicit any discernible impact on tumor growth (Fig. 4C). These results are contradictory to our initial assumptions. Subsequently, we proceeded to explore the potential influence of rmIL-33 treatment on the infiltration of ILC2s within the TME. Our experimental results revealed a significant increase in ILC2s infiltration in the rmIL-33-treated group of tumor-bearing mice, in comparison to the control group (Fig. 4D–F). The lack of discernible antitumor effect observed in response to rmIL-33 administration led us to postulate that rmIL-33 may selectively induce expansion of specific subsets of ILC2s. We found that rmIL-33 mainly promoted the infiltration of nILC2s, which was defined as ST2^+^ KLRG1^−^ ILC2s (Fig. 4G, H), on which ST2 is the receptor of IL-33. Regarding T cell-mediated antitumor immune responses, despite the absence of consistent alterations in the proportions of total and intra-tumoral CD4+ T cells and CD8+ T cells following rmIL-33 treatment (Fig. 5A–D), the possibility of functional modulation cannot be ruled out. This is supported by the enhanced IFN-γ production capacity and upregulation of PD-1 observed in intra-tumoral CD4^+^ T cells and CD8_+_ T cells (Fig. 5E–H). Taken together, these findings suggest that the accumulation of IL-33-induced nILC2s subsets in tumor tissues does not effectively impede tumor growth. However, this phenomenon is accompanied by the upregulation of PD-1 and IFN-γ expression in T cells.

### PD-1 blocked augments iILC2s—mediated anti-tumor immunity

**Fig. 6:**
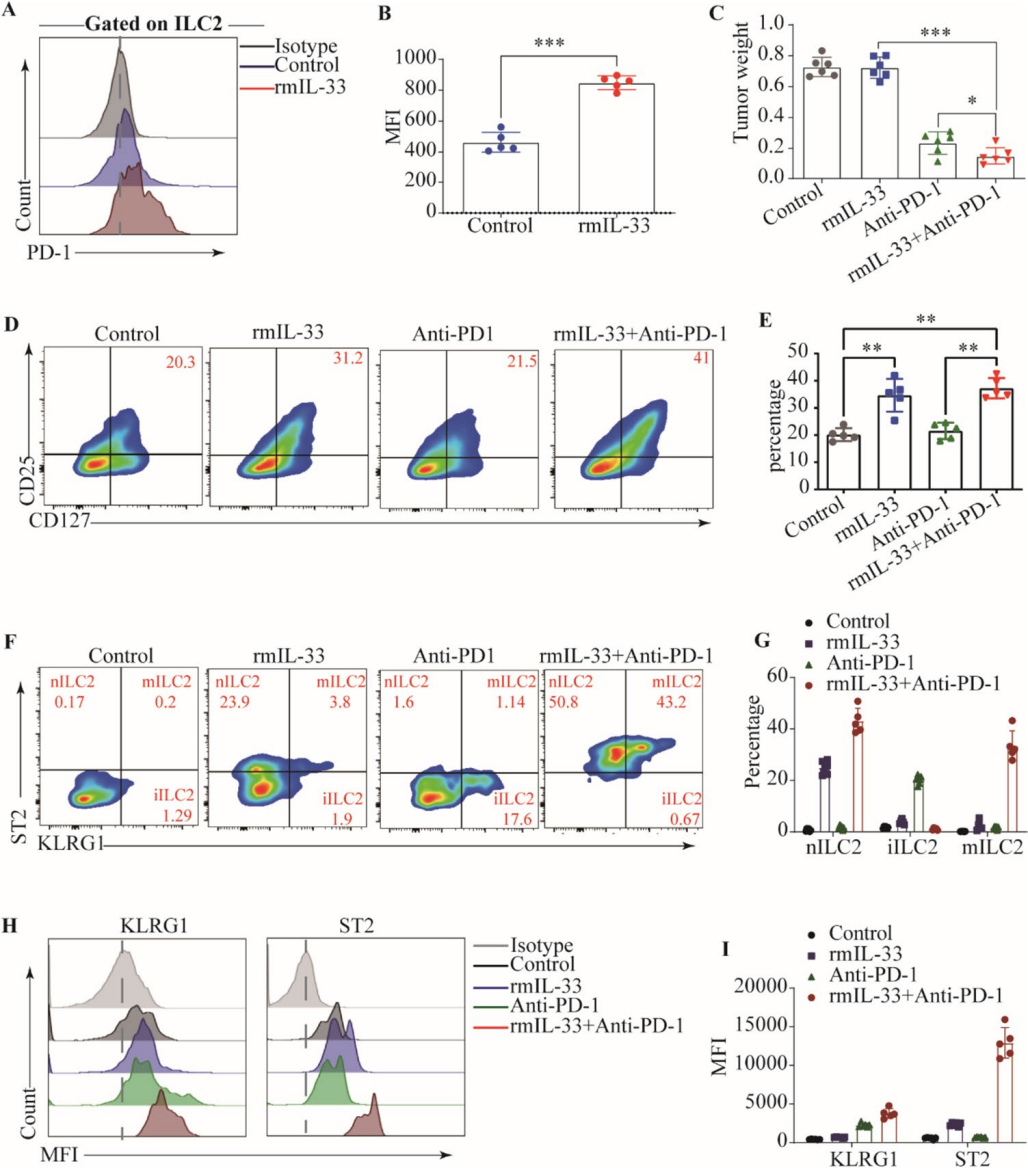
PD-1 blocked augments ILC2s-mediated anti-tumor immunity.** A** PD-1 staining in the different cohorts of ILC2s was analyzed by flow cytometry. **B** PD-1 mean fluorescence intensity (MFI) of ILC2s. **C**, Tumor weights of tumors in tumor-bearing mice post of different treatments. Gating (**D**) and frequency (**E**) of ILC2s in tumor tissues from tumor-bearing mice post of different treatment. Gating (**F**) and frequencies (**G**) of ILC2s sunsets in tumor tissues from tumor-bearing mice post of different treatments. Staining (**H**) and MFI (**I)** of KLRG1 and ST2 of ILC2s in tumor tissues from tumor-bearing mice post of different treatments were analyzed by flow cytometry. Data were pooled from ≥ 2 independent experiments with *n* = 5/group; n and data points denote individual mice analyzed separately. *P* values were determined by unpaired t test (**B**, **F**), two-way analysis with multiple comparisons (**C**, **E**). ***P < 0.001, **P < 0.01, *P < 0.05

Previous studies have shown that ILC2s express PD-1 [25, 26], considering the observed increase in PD-1 expression on T cells following rmIL-33 activation, we proceeded to examine PD-1 expression on ILC2s upon stimulation. Notably, we observed a significant elevation in PD-1 expression on ILC2s cells in mice treated with rmIL-33 (Fig. 6A, B). These findings have driven us to explore the potential of simultaneous activation of ILC2s and disinhibition in TME for cancer therapy. Our investigations revealed that the combined administration of rmIL-33 and PD-1 blockade exhibited a synergistic effect in activating ILC2s, resulting in enhanced antitumor efficacy. Notably, this combined approach demonstrated superior tumor control compared to treatment with anti-PD-1 alone (Fig. 6C, Supplementary Fig. 6), as well as expanded ILC2s in tumors (Fig. 6D, E, Supplementary Fig. 7). In tumor-bearing mice treated with rmIL-33 and anti-PD-1, ILC2s exhibited distinct phenotypic alterations, characterized by heightened expression of ST2 and KLRG1 (Fig. 6F, G). Specifically, the combined treatment induced a unique subset of ILC2s referred to as mILC2s, which displayed these enhanced surface marker expressions. Our findings suggest that rmIL-33 primarily enhances the immune response mediated by nILC2s, while blockade of PD-1 augments the anti-tumor immune potential of iILC2s (Fig. 6H, I). In summary, these findings collectively indicate that the concurrent administration of IL-33 and PD-1 blockade can significantly potentiate the immunotherapeutic efficacy of anti-PD-1 treatment.

## Discussion

Tumor immune infiltration exhibits heterogeneity, and the makeup and functionality of infiltrating immune cells play a significant role in determining patient prognosis. Numerous research teams have highlighted the significance of ILC2s in both anti-tumor and pro-tumor immune responses across various mouse and human malignancies. However, the regulatory mechanisms governing the functional aspects of ILC2s in tumor immunity remain largely unexplored. Previous study has demonstrated that ILC2s secrete the characteristic cytokines IL-13 and IL-4, which facilitate tumor progression by facilitating the recruitment of monocyte-derived myeloid suppressor cells (MDSCs), which plays a crucial role in suppressing anti-tumor immune responses [27]. In contrast, Moral et al. have presented evidence indicating that augmenting the population of ILC2s in human PDAC positively impacts patient survival rates. This improvement is attributed to the enhanced activation of CD8^+^ T cells and subsequent reinforcement of anti-tumor immune responses [28]. Schuijs et al. have documented that IL-5, derived from ILC2s, stimulates eosinophils and subsequently suppresses Th1-mediated anti-tumor immunity, which ultimately promotes lung cancer metastasis and escalates mortality rates [29]. Conversely, Wagner et al. corroborated the notion that activated ILC2s trigger an upregulation of IL-5, thereby enhancing the cytotoxic potential of eosinophils against tumor growth and lung metastasis [30]. These discrepant findings have prompted us to delve deeper into the heterogeneity of this phenomenon.

Several studies have demonstrated that the population of ILC2s can be further classified into three distinct subsets, namely nILC2s, iILC2s, and mILC2s. Interestingly, it has been observed that the absence of PD-1 expression leads to an increase in both the quantity and functional capacity of iILC2s [31]. In this study, we present evidence indicating that there is an increased presence of infiltrating ILC2s in tumor tissues as compared to normal tissues. However, intriguingly, the ILC2s within the TME were found to be in a quiescent state, characterized by the absence of ST2 and KLRG1 expression (ST2^−^KLRG1^−^ ILC2s). Through the utilization of IL-33^−/−^ mice, we demonstrate that the loss of host-derived IL-33 promotes tumor growth and induces alterations in the infiltration patterns of ILC2s, CD4^+^ T cells, and CD8^+^ T cells. These findings furnish compelling evidence corroborating the hypothesis that the antineoplastic effects exerted by IL-33 are intricately intertwined with the functional capacities of ILC2s and T cells. Tumor-bearing mice treated with rmIL-33 exhibited no significant inhibition in tumor growth. Subsequent investigations revealed that the enhanced ILC2s population displayed the nILC2s phenotype (ST2^+^KLRG1^−^ ILC2s). Additionally, rmIL-33 administration augmented the cytokine capacity of intra-tumoral CD4^+^ T cells and CD8^+^ T cells, leading to an upregulation of PD-1 expression. Interestingly, ILC2s were found to express the immune checkpoint PD-1, which was further upregulated upon activation by IL-33. These findings suggest that ILC2s may contribute to the effectiveness of PD-1 pathway blockade in cancer treatment. However, their potential for exploitation in immune checkpoint blockade therapy remains uncertain. The co-administration of IL-33 and PD-1 blockade demonstrates an inhibitory effect on tumor growth, accompanied by an augmented presence of intra-tumoral ILC2s, particularly the mILC2s phenotype (ST2^+^KLRG^+^ ILC2s). Furthermore, our investigation revealed a reduced infiltration of Lin^−^KLRG1^+^ cells in human lung cancer tissues in comparison to para-carcinoma tissues.

In conclusion, our findings shed light on a mechanism for enhancing the functionality of ILC2s, emphasizing the importance of considering distinct subsets of ILC2s. Moreover, our results indicate that immune checkpoint blockade alone may not suffice to achieve optimal outcomes. This underscores the need for a comprehensive understanding of the heterogeneous responses to checkpoint blockade and the development of refined methodologies for identifying ILC2s in human cancers. Such efforts will expedite investigations into the prognostic and predictive capacities of ILC2s.

### Supplementary Information

Below is the link to the electronic supplementary material.

Supplementary file1 (DOCX 3231 KB)

## Data Availability

The authors confirm that the data used and/or analysed during the current study are available from the corresponding author on reasonable request.
