# Supplementary material for: Combination of IL-33 with PD-1 blockade augment mILC2s-mediated anti-tumor immunity
Source: Cancer Immunol Immunother. 2024 Mar 2;73(4):65. doi: 10.1007/s00262-023-03580-7 (PMC10908611; doi:10.1007/s00262-023-03580-7)
Supplement: Supplementary file 1 — Supplementary file1 (DOCX 3231 KB) [file 262_2023_3580_MOESM1_ESM.docx]

**Supporting Information**


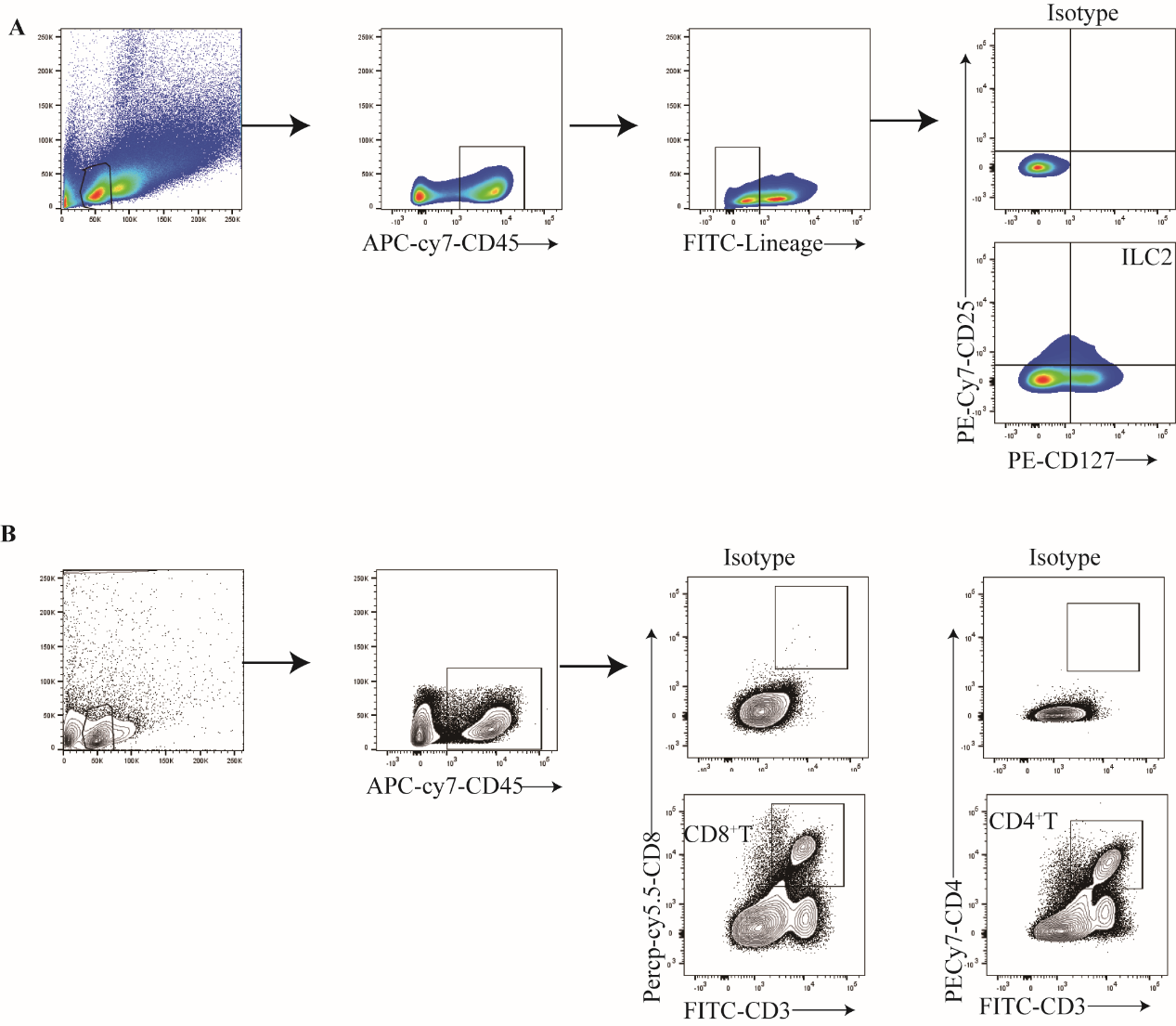
 **Supplementary Fig. 1** **Gating strategy.** **A,** Gating and phenotype of ILC2s. **B,** Gating and phenotype of CD4^+^T cell and CD8^+^T cell.


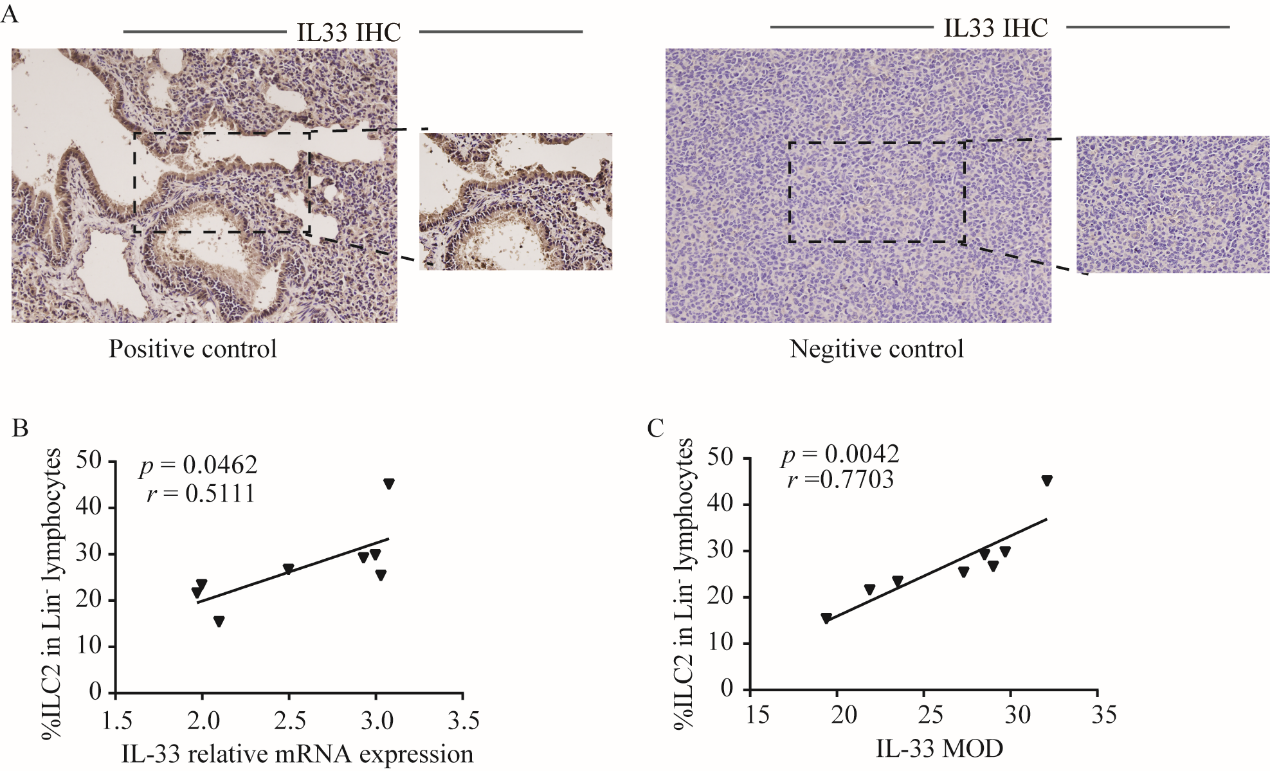


**Supplementary Fig. 2** **A**, Positive and negative control of IL-33 expression in tissues. Correlations between the levels of IL-33 mRNA expression (**B**) and IL-33 MOD (**C**) with the percentage of ILC2s in tumor tissues from lung adenocarcinoma tumor-bearing mice.


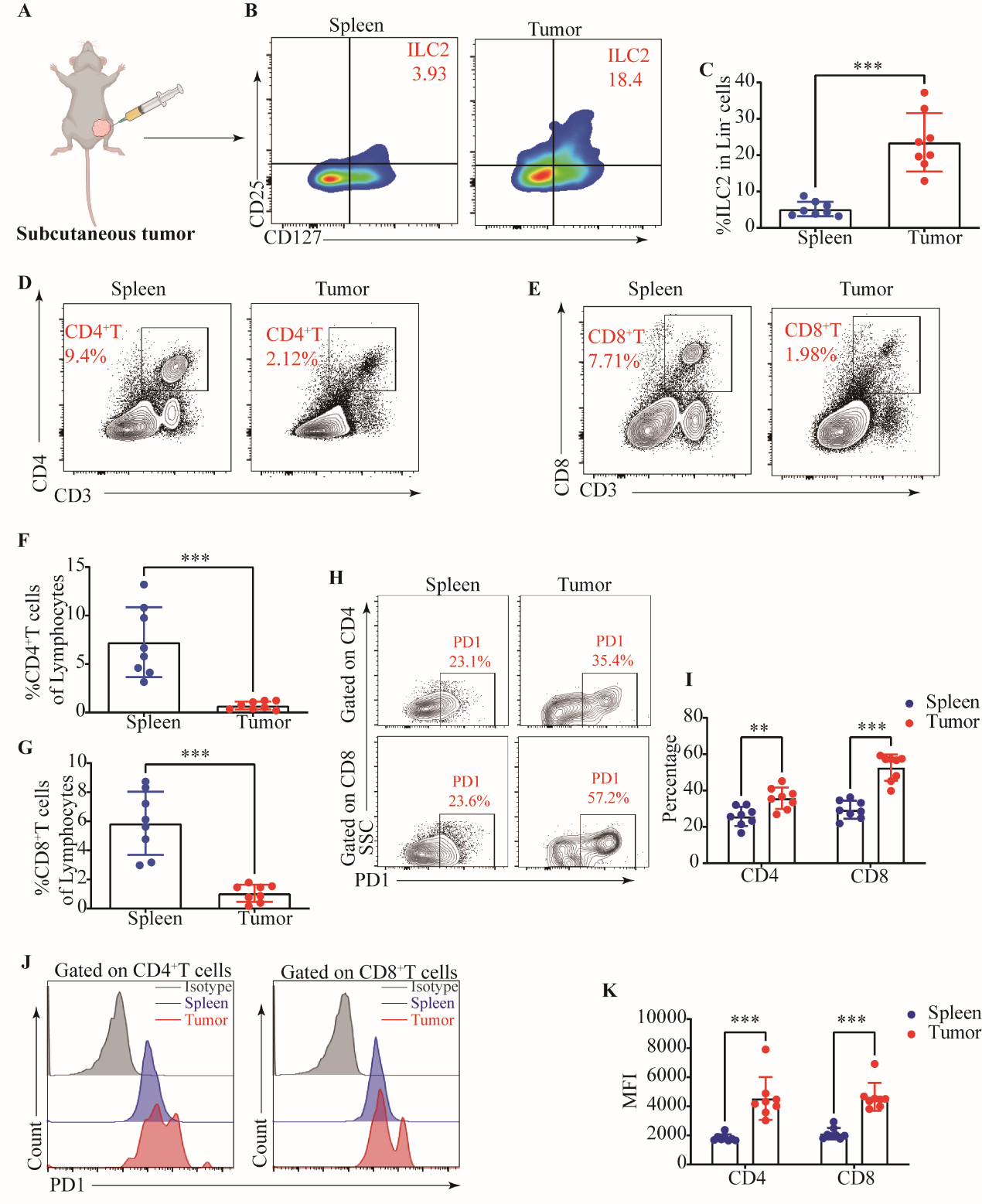


**Supplementary Fig. 3 ILC2s infiltrating in tumor tissues in subcutaneous LUAD tumor. A,** Subcutaneous LUAD tumor model. Gating (**B**) and frequency (**C**) of ILC2s in subcutaneous LUAD tumor-bearing mouse. Gating and phenotype of CD4^+^T cell (**D**), and CD8^+^T cell (**E**) in spleen and tumor tissue. Frequency of CD4^+^T cell (**F**), and CD8^+^T cell (**G**) in spleen and tumor tissue. Gating (**H**) and frequency (**I**) of PD1 in CD4^+^T cell and CD8^+^T cell in spleen and tumor tissue. Staining (**J**) and MFI (**K**) of PD-1 in CD4^+^T cell and CD8^+^T in spleen and tumor tissue. Data were pooled from ≥2 independent experiments with *n* = 8/group; n and data points denote individual mice analyzed separately. *P* values were determined by unpaired t test (**C**, **F, G**), two-way analysis with multiple comparisons (**I**, **K**). ****P* < 0.001, ***P* < 0.01.


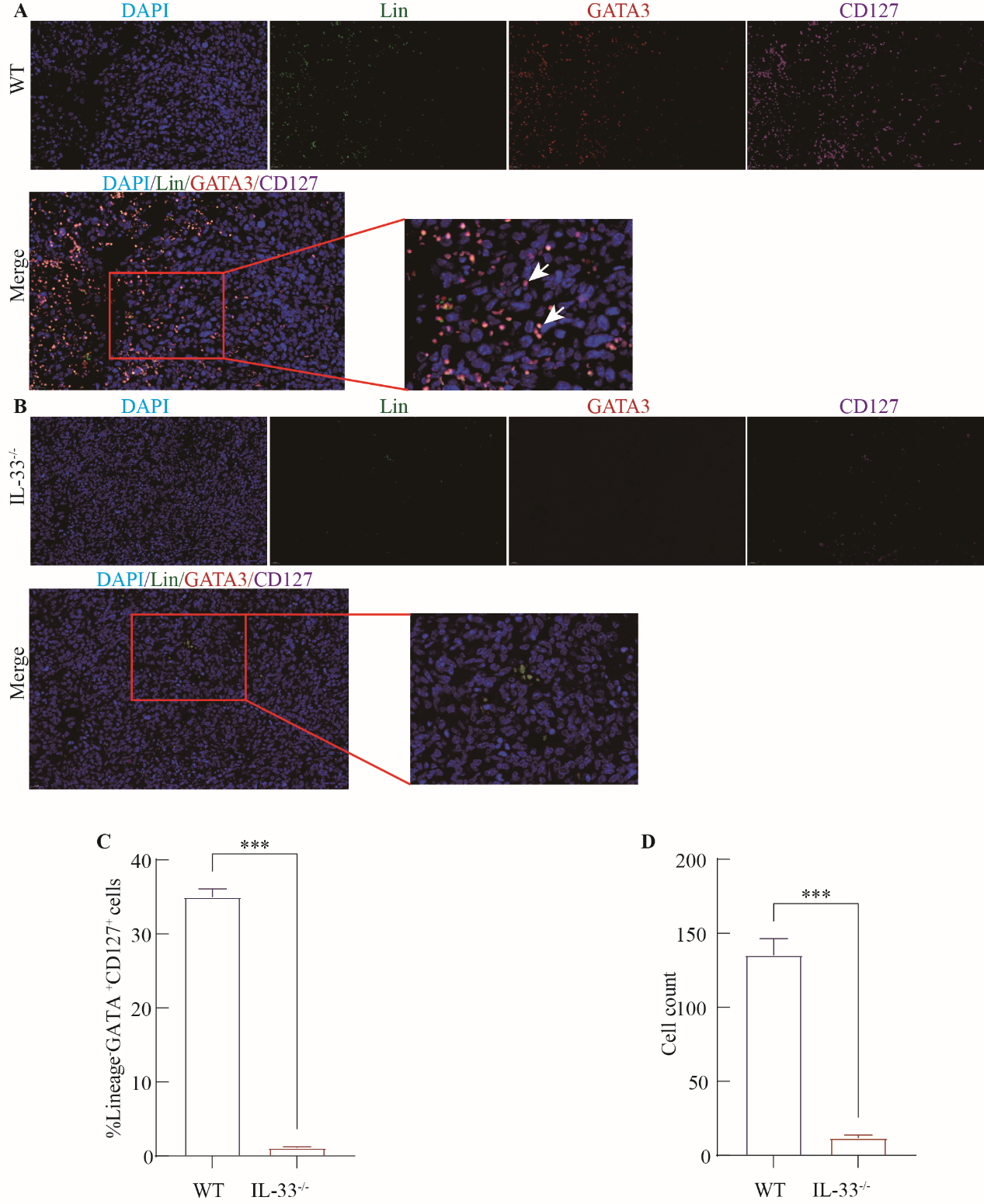


**Supplementary Fig. 4 Murine tumor-derived ILC2s were detected by immunofluorescence. A**, Murine tumor-derived ILC2s in tumor tissue of WT mice. **B**, Murine tumor-derived ILC2s in tumor tissue of IL-33^-/-^ mice. **C**, ILC2s frequency in tumor tissue of WT and IL-33^-/-^mice. **D**, ILC2s cell number in tumor tissue of WT and IL-33^-/-^mice. Data were pooled from ≥2 independent experiments; *n* = 5/group. *P* values were determined by unpaired t test. ****P* < 0.001.


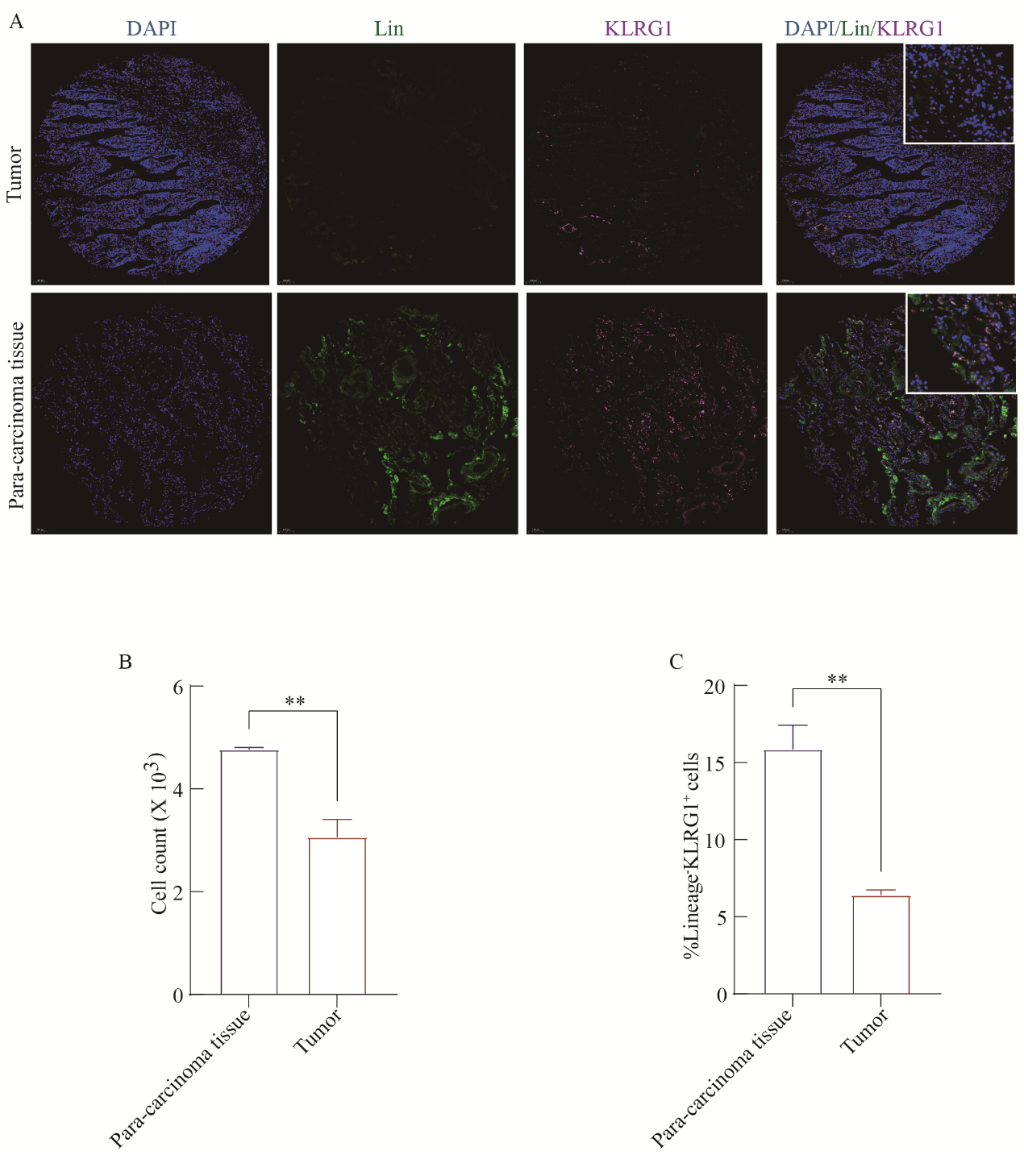
 **Supplementary Fig. 5 Human Lin^-^KLRG1^+^ cell was detected by IF. A**, Human Lin^-^KLRG1^+^ cell in tumor tissue and Para-carcinoma tissue. **B**, Lineage^-^KLRG1^+^ cell count in tumor tissue and Para-carcinoma tissue. **C**, Lineage^-^KLRG1^+^ cell frequency in tumor tissue and Para-carcinoma tissue. Data were pooled from 3 independent experiments; *P* values were determined by unpaired t test. ***P* < 0.01.


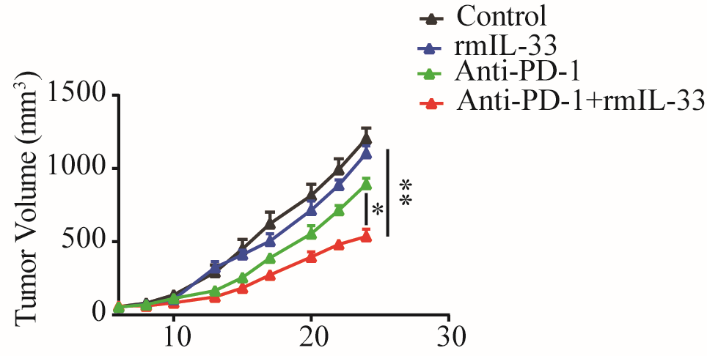


**Supplementary Fig. 6** The tumor growth curves of tumor-bearing mice post of different treatments.


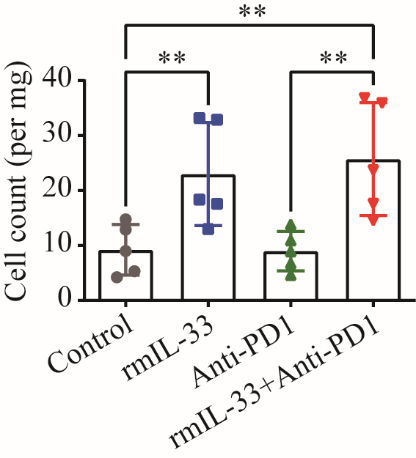


**Supplementary Fig. 7** Numbers of ILC2s in tumor tissues from tumor-bearing mice post of different treatments.
